# Crosstalk between microglia and patient-derived glioblastoma cells inhibit invasion in a three-dimensional gelatin hydrogel model

**DOI:** 10.1186/s12974-020-02026-6

**Published:** 2020-11-18

**Authors:** Jee-Wei Emily Chen, Jan Lumibao, Sarah Leary, Jann N. Sarkaria, Andrew J. Steelman, H. Rex Gaskins, Brendan A. C. Harley

**Affiliations:** 1 Department of Chemical & Biomolecular Engineering, University of Illinois at Urbana-Champaign, Urbana, IL 61801 USA; 2 Carl R. Woese Institute for Genomic Biology, University of Illinois at Urbana-Champaign, Urbana, IL 61801 USA; 3 Department of Materials Science and Engineering, University of Illinois at Urbana-Champaign, Urbana, IL 61801 USA; 4 Division of Nutritional Sciences, University of Illinois at Urbana-Champaign, Urbana, IL 61801 USA; 5 Current Address: Salk Institute for Biological Studies, La Jolla, CA USA; 6 Department of Chemistry, University of Illinois at Urbana-Champaign, Urbana, IL 61801 USA; 7 Department of Radiation Oncology, Mayo Clinic, Rochester, MN USA; 8 Department of Animal Sciences, University of Illinois at Urbana-Champaign, 110 Roger Adams Laboratory, 600 S. Mathews Ave, Urbana, IL 61801 USA; 9 Cancer Center at Illinois, University of Illinois at Urbana-Champaign, 110 Roger Adams Laboratory, 600 S. Mathews Ave, Urbana, IL 61801 USA

**Keywords:** Glioblastoma, Microglia, Invasion, Hydrogel

## Abstract

**Background:**

Glioblastoma is the most common and deadly form of primary brain cancer, accounting for more than 13,000 new diagnoses annually in the USA alone. Microglia are the innate immune cells within the central nervous system, acting as a front-line defense against injuries and inflammation via a process that involves transformation from a quiescent to an activated phenotype. Crosstalk between GBM cells and microglia represents an important axis to consider in the development of tissue engineering platforms to examine pathophysiological processes underlying GBM progression and therapy.

**Methods:**

This work used a brain-mimetic hydrogel system to study patient-derived glioblastoma specimens and their interactions with microglia. Here, glioblastoma cells were either cultured alone in 3D hydrogels or in co-culture with microglia in a manner that allowed secretome-based signaling but prevented direct GBM-microglia contact. Patterns of GBM cell invasion were quantified using a three-dimensional spheroid assay. Secretome and transcriptome (via RNAseq) were used to profile the consequences of GBM-microglia interactions.

**Results:**

Microglia displayed an activated phenotype as a result of GBM crosstalk. Three-dimensional migration patterns of patient-derived glioblastoma cells showed invasion was significantly decreased in response to microglia paracrine signaling. Potential molecular mechanisms underlying with this phenotype were identified from bioinformatic analysis of secretome and RNAseq data.

**Conclusion:**

The data demonstrate a tissue engineered hydrogel platform can be used to investigate crosstalk between immune cells of the tumor microenvironment related to GBM progression. Such multi-dimensional models may provide valuable insight to inform therapeutic innovations to improve GBM treatment.

**Supplementary Information:**

The online version contains supplementary material available at 10.1186/s12974-020-02026-6.

## Introduction

Glioblastoma (GBM) is the most common and deadly form of central nervous system cancer [1, 2]. In the USA, it is estimated that more than 13,000 patients are diagnosed with GBM annually. Unlike many other cancers, GBM rarely metastasizes to a secondary organ, but instead diffusely infiltrates throughout the brain. The current standard of care for treating GBM consists of maximal surgical resection, radiotherapy, and concomitant and adjuvant chemotherapy with temozolomide [1, 3, 4]. Despite this aggressive treatment strategy, GBM tumors commonly recur with a median survival of less than 18 months, and fewer than 5% of patients surviving to 5 years [5–11]. A central focus for improving GBM therapy is developing new tools to understand pathophysiological processes driving GBM invasion of the brain. Improved therapy will likely require both an improved understanding of which cells within the heterogeneous cell population of GBM tumors invade surrounding tissues, and the extent to which cell-cell crosstalk within heterogeneous cell cohorts contribute to GBM invasion and mortality [12–18].

Microglia (MG) are resident immune cells of the CNS [19, 20]. In healthy individuals, microglia constantly survey their surroundings and maintain tissue homeostasis by removing apoptotic cells and promoting neuro-network generation [20–22]. Studies of the GBM tumor microenvironment have demonstrated that infiltrative and resident immune cells, such as microglia, may comprise up to a third of the solid tumor mass [20–22]. Morphologically, quiescent microglia typically exhibit a ramified (branching and elongated) shape. Upon stimulation in response to inflammation, disease, or tumor growth, microglia cell processes become hypertrophic and, in some cases, retract causing the cell to take on an ameboid appearance. While the number and phenotype of immune cells have been associated with patient prognosis [19, 23–27], detailed analysis of crosstalk between GBM cells and microglia are difficult to evaluate in vivo. Thus, there is a need for an experimental platform to rigorously investigate interactions between GBM cells and microglia, as well as to identify factors associated with microglia-GBM crosstalk that may alter GBM cell invasion and therapeutic response. Cancer tissue engineering platforms that integrate biomaterial mimics of the tumor microenvironment with primary cells and biomolecules are increasingly used to investigate pathophysiological processes difficult to examine in vivo [28, 29].

We previously developed a gelatin-based hydrogel model platform to investigate pathophysiological processes underlying GBM cell invasion and therapeutic response. Notably, we observed that biophysical (hyaluronan content and molecular weight) and metabolic (hypoxia) transitions in the GBM tumor microenvironment both significantly alter GBM invasion [30–32]. More recently, we adapted this system to profile cytokine-based crosstalk between cells within the GBM tumor microenvironment, identifying secreted factors generated by an artificial perivascular niche that can accelerate GBM cell invasion [33]. The objective of the present study was to adapt this established hydrogel platform and cytokine analysis protocols to examine the effects of microglia within the GBM tumor microenvironment on GBM gene expression and invasiveness using patient-derived GBM specimens that maintain patient-specific morphologic and molecular phenotypes [34, 35].

## Materials and methods

### Cell culture

#### Human GBM cells

Patient-derived GBM cells (PDCs) were obtained from Mayo Clinic (Rochester, Minnesota). All specimens used in this study (GBM12 and GBM39) were derived from tumors from different patients then maintained as patient-derived xenografts in nude mice [34, 35]. All patients consented to the use of their tumor tissue in support of this research, and the use of the patient tissues received prior institutional review board authorization. GBM12 exhibits overexpressed epidermal growth factor receptor (*EGFR*^*OE*^) and displays medium invasive potential in vivo. GBM39 possesses an *EGFR* variant III mutation (*EGFR*^*vIII*^) and displays low invasive potential in vivo [34, 35]. GBM PDCs were established in Dulbecco’s modified Eagle’s medium (DMEM; Gibco, MD) supplemented with 10% fetal bovine serum (FBS; Atlanta Biologicals, Atlanta, GA) and 1% penicillin/streptomycin (Lonza, Basel, Switzerland) at 37 °C in a 5% CO_2_ environment. PDCs were shipped by overnight courier and seeded into hydrogel cultures immediately upon arrival.

#### Human microglia cell line

HMC3 microglia cells (ATCC®CRL-3304, ATCC) were cultured in DMEM supplemented with 10% FBS and 1% P/S. Cells were incubated at 37 °C in 5% CO_2_ and passaged upon reaching confluence.

#### Primary mouse microglial cultures

All animal care protocols were in accordance with NIH Guidelines for Care and Use of Laboratory Animals and were approved by the University of Illinois Laboratory Animal Care and Use Committee. Both male and female C57BL/6J mice (The Jackson Laboratory, Bar Harbor, ME) were used to obtain primary microglial cultures as described previously [36]. In brief, neonatal (P1–2) mouse pups were decapitated with scissors, the brains were extracted, and the meninges removed under a dissection microscope (Leica, Wetzlar, Germany). Brain tissues were pooled from entire litters, dissociated in Accutase (Thermo Fisher Scientific, Waltham, MA) followed by washing and removal of excess debris. Cells were seeded onto poly-d-lysine-coated (Sigma-Aldrich, St. Louis, MO) T75-flasks. After approximately eight days of culture, the flasks were shaken at 37 °C for 1 h at 170 rpm in an orbital shaker (Max Q 4000; Thermo Fisher Scientific). The supernatant containing microglia was then collected [36].

### Methacrylated gelatin hydrogel fabrication and characterization

#### Synthesis and fabrication

Methacrylamide-functionalized gelatin (GelMA) macromers and GelMA hydrogels were synthesized and prepared as previously described [30, 37]. GelMA degree of functionalization was determined by ^1^H NMR spectroscopy (~ 50% degree of functionalization).

#### Hydrogel characterization

The compressive modulus of GelMA hydrogels was measured using an Instron 5943 mechanical tester [31]. Hydrogels were tested under unconfined compression at the rate of 0.1 mm/min, with their Young’s modulus obtained from the linear region of the stress-strain curve (2.5–17.5% strain).

#### Cell number determination

Cell-containing hydrogels were made similarly but with addition of cell suspensions (5000 cells per 25 μL hydrogel) or cell spheroids (5000 cells per spheroid per 25 μL hydrogel) to the pre-polymer solution prior to being placed into Teflon molds (0.2 mm thick, 5 mm radius) and then photopolymerized.

*Hydrogel identification*: To distinguish hydrogels containing PDCs versus microglia, all hydrogel specimens containing microglia were cut into half-disks before being placed into culture while GBM seeded hydrogels were maintained as full disks.

### Protein Isolation and Western Blotting

Proteins from HMC3 microglia cultured in hydrogels (cell suspensions) were isolated using protocols described previously [30–32]. Western blots (2 μg per lane) were probed with primary antibodies specific for CD68 [38] (ab213363, 1/500 in blocking buffer; Abcam, Cambridge, UK) or β-actin (4967S, 1/1000 in blocking buffer; Cell Signaling Technology, Danvers, MA), stained via secondary antibody (7074S, 1/2500 in TBST; Cell Signaling Technology), then imaged via an Image Quant LAS 4010 chemiluminescence imager (GE Healthcare, Chicago, IL). Band intensities were quantified using ImageJ and normalized to β-actin intensities.

### Immunofluorescence staining and imaging

Image-iT™ Fixative Solution (Invitrogen, Carlsbad, CA) was used to fix HMC3 microglia seeded hydrogels (cell suspensions). Cells were permeabilized with 0.1% Triton X-100 (Sigma-Aldrich) in PBS and stained with Alexa Fluor™ 488 Phalloidin (Invitrogen) for F-actin and Hoechst 33342 (Invitrogen) for nuclei following the manufacturer’s protocol. Stained samples were imaged using a Zeiss LSM 700 confocal microscope.

### RNA extraction and quality analysis

Total RNA of GBM cell-seeded hydrogels (cell suspensions) was extracted using an RNeasy Plant Mini Kit (Qiagen, Hilden, Germany) with an additional step using an RNase-free DNase set (Qiagen) for DNase digestion. RNA integrity number (RIN) was determined using an Agilent 2100 bioanalyzer with all samples exhibiting a RIN > 8.

### RNAseq analysis

Libraries for RNA sequencing were prepared using the TruSeq Stranded mRNAseq Sample Prep Kit (Illumina, San Diego, CA). The libraries were quantitated by qPCR and sequenced on one lane for 101 cycles from one end of the fragments on a NovaSeq 6000 (Illumina) using a NovaSeq SP reagent kit and yielded 400 to 500 million single reads per lane. Library quality check was done using FastQC (version 0.11.8). Salmon version 0.13.1 was used to quasi-map reads to the transcriptome and quantify the abundance of each transcript. Filtering was set with the threshold of 0.5 counts per million and resulted in 15,901 genes to be analyzed for differential expression that contained > 99.5% of the reads. After filtering, trimmed mean of M values (TMM) normalization in the edgeR package was performed and log2-based count per million values (logCPM) were calculated [39–41]. Differential gene expression analysis was performed using the limma-trend method. Multiple testing correction was done using the false discovery rate (FDR) method [42–44]. Functional annotation was performed, gene ontology (GO) KEGG pathways were identified using an overrepresentation test [45]. The 50 genes with the highest fold-changes were subsequently examined in Cytoscape using the application iRegulon to predict transcriptional regulators [46, 47]. Putative transcription factors or motifs with a normalized enrichment score larger than three (NES > 3) is considered to be potential regulators.

### Spheroid invasion assay

We have previously reported a robust, three-dimensional assay to quantify GBM cell invasion in three-dimensional hydrogels [48–50]; GBM cell invasion is reported as total radial invasion distance or as a normalized value such as the average radius fold change (mean radius of the invasion front of GBM cells from a GBM spheroid versus the starting radius of the spheroid). Briefly, GBM12 PDCs were counted and resuspended into 5000 cells/200 μL media per well and distributed to Corning spheroid microplates. Plates were centrifuged for 100×*g* for 1 min to assist spheroid formation then placed into incubator (37 ^o^C, 5% CO_2_) for 24 h. Plates were then incubated for additional 24 h with horizontal shaking at 60 rpm. Spheroids were then transferred and mixed with pre-polymerized GelMA solution and photopolymerized into hydrogels. Spheroid images were acquired using a Leica DMI 400B florescence microscope (Leica, Germany) at days 0 (immediately upon seeding), 1, 3, 5, and 7. Invasion was then quantified via ImageJ and invasion distance reported as fold change of their average radius compared to day 0 as described previously [30, 31].

### Quantification of cell number

GBM12 cell proliferation in cell suspension hydrogels was determined using a commercial Vybrant® MTT Cell Proliferation Assay Kit (Invitrogen) adapted from the manufacturer’s protocol as described previously [30, 32, 51].

### Secretome profiling

Cell culture media was collected from the following group: GBM-MG co-cultures (co-culture), GBM cells alone (GBM single), or MG cells alone (MG single). The media were spun down (300×*g*, 10 min) to remove any debris. As an additional control, we created a 1:1 mixture of GBM single and MG single media (Mix) that would not account for any GBM-MG crosstalk mediated shifts in secretome. The secretome for each specimen was profiled using a Proteome Profiler^TM^ Human Angiogenesis Array Kit (Ary007, R&D Systems, Minneapolis, MN) following the manufacturer’s protocol. Blots were imaged using an Image Quant LAS 4010 chemiluminescence imager (GE Healthcare). Dot intensities were quantified with the ImageJ macros toolset Protein Array Analyzer (Table S1). Data was first normalized by dividing the pixel intensity for each blot by the average positive control pixel intensity (on each membrane). We calculated intensity fold change between co-culture and mix groups, identifying factors that displayed a larger than 1.5-fold change; targets showing > 0.75-fold change relative to positive reference spot intensities was plotted.

### Statistical analysis

Statistical analysis for Western blot was performed via *t* test. Analysis of MTT was performed via one-way ANOVA followed by Tukey’s HSD post hoc test. Analyses of invasion were performed via two-way ANOVA followed by Tukey’s HSD post hoc test. Significance level was set at *p* < 0.05 unless stated otherwise (*p* < 0.01 and *p* < 0.0001). A minimum of *n* = 3 samples was used for all analyses and specified in each result section. Error bars are plotted as the standard error.

## Results

### Co-culture system assembly and mechanical testing of GelMA hydrogel

**Fig. 1:**
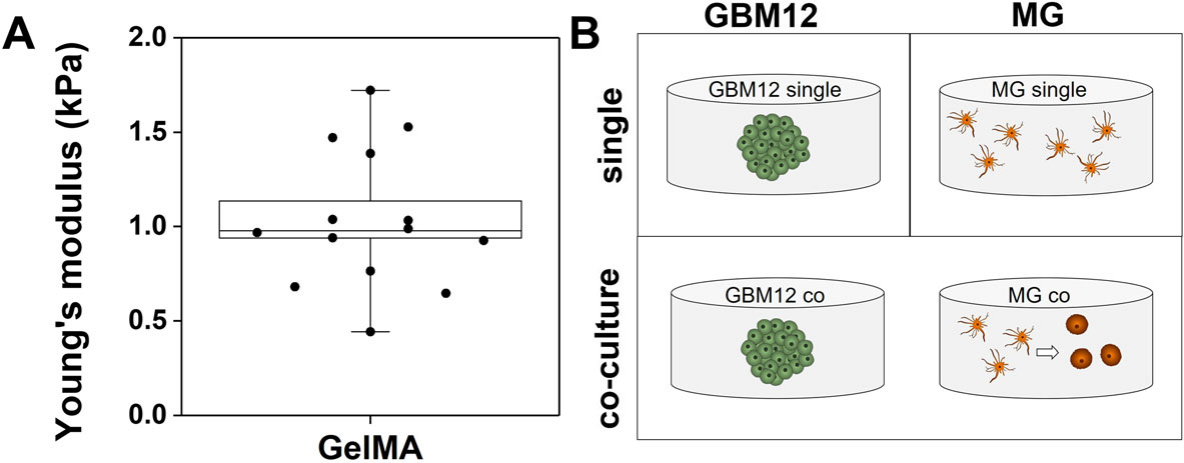
GelMA hydrogel biophysical parameters and experimental schematic. **a** Mechanical testing results of GelMA hydrogel confirmed its physiologically relevant Young’s modulus (1.04 ± 0.10 kPa; *n* = 14). **b** Schematic representation of the culture system, that either has a single type of cell-seeded hydrogel disk per well (*GBM single*, *MG single*) or contains two distinct hydrogel disks (GBM-MG *co-culture*). GBM-MG *co-culture* allows soluble signaling without physical GBM-MG contact

We have previously developed a family of gelatin hydrogels to investigate pathophysiological processes underlying glioblastoma invasion and progression. Extensive biophysical performance data has previously been reported including network architecture and mechanical performance [52, 53], water and small-molecule diffusivity [54–56], and the capacity to support growth and phenotypic studies of primary glioblastoma cells and cells from the neurovascular unit [57, 58]. For this project, GBM and MG cells were maintained in three-dimensional culture in a 4 wt% methacrylamide-functionalized gelatin (GelMA) that exhibited a physiologically relevant Young’s modulus (1.04 ± 0.10 kPa; *n* = 14; Fig. 1a) [59, 60]. For this study, individual (*GBM single*, *MG single*) cultures were generated as either GBM

or MG-seeded hydrogel disks in separate culture wells. Alternatively, GBM and MG seeded hydrogel disks were cultured together in the same well (*co-culture*), allowing exchange of secreted factors between hydrogel disks but not allowing direct cell-cell contact (Fig. 1b).

### Microglia become activated when co-cultured with GBM cells

**Fig. 2:**
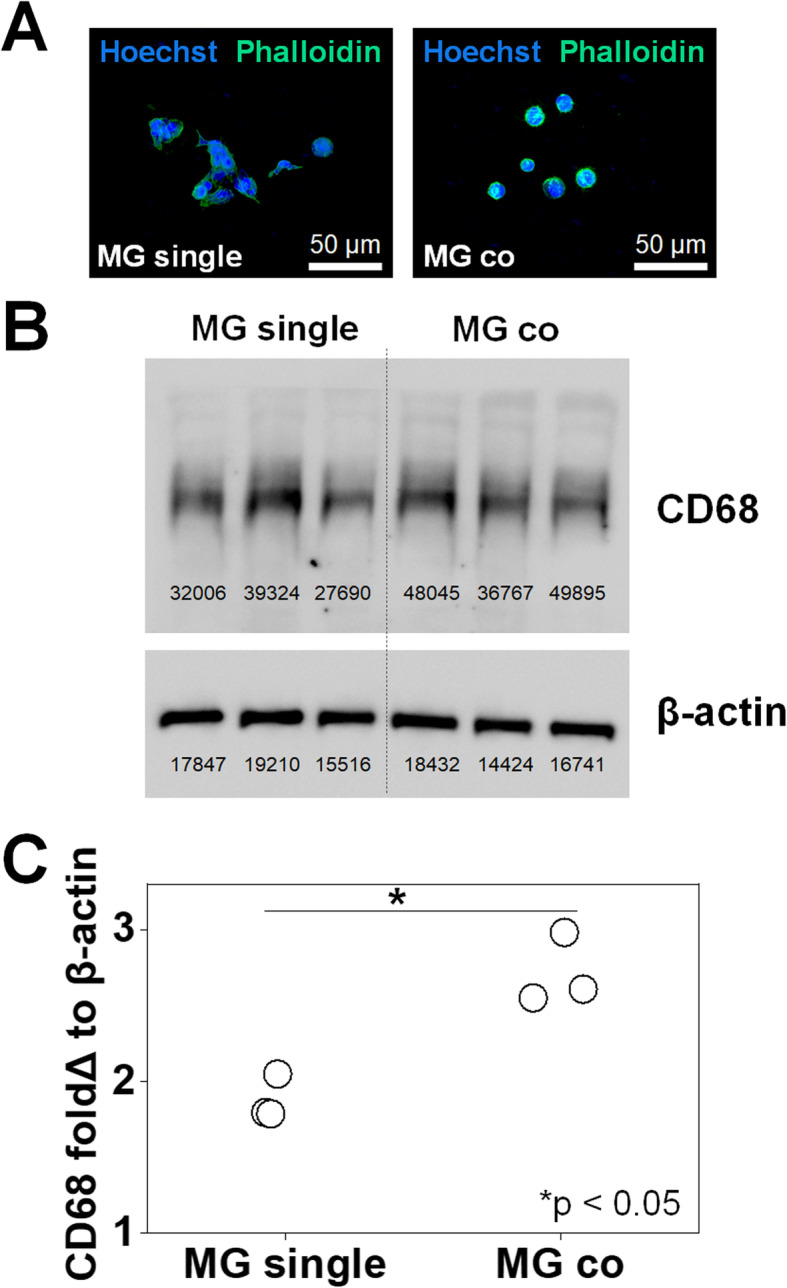
Verification of microglia activation via morphological and protein expression analyses. **a** Immunofluorescence staining of Alexa488Phalloidin^+^ actin (green) showing HMC3 microglia morphology in *MG single* (elongated quiescent) or GBM-MG *co-culture* (ameboid, activated). Also, Hoechst 33342^+^ nuclei stained blue. **b** Western blot staining CD68 and β-actin in triplicate. MG single on the left and MG co-culture on the right. **c** CD68 protein expression levels (*n* = 3) in microglia increased as a result of GBM co-culture. *p < 0.05

Microglia displayed significant morphological changes as a result of GBM-MG crosstalk (Fig. 2). HMC3 microglia cultured individually in GelMA hydrogels (*MG single*) exhibited the characteristic elongated shape of quiescent MG. However, in response to co-culture with GBM12-seeded hydrogels (GBM-MG *co-culture*), MG adopted a rounded ameboid shape associated with activation (Fig. 2a). To confirm this shift in activation status, changes in the expression of the lysosomal protein CD68 were examined via Western blot (*n* = 3). While CD68 is normally expressed at low levels in quiescent microglia, CD68 expression was increased in microglia co-cultured with GBM cells (Fig. 2b, c). Raw image files of Western blot analyses are provided in Figures S1 and S2.

### Soluble factors produced by microglia altered GBM transcriptomic profiles

**Fig. 3:**
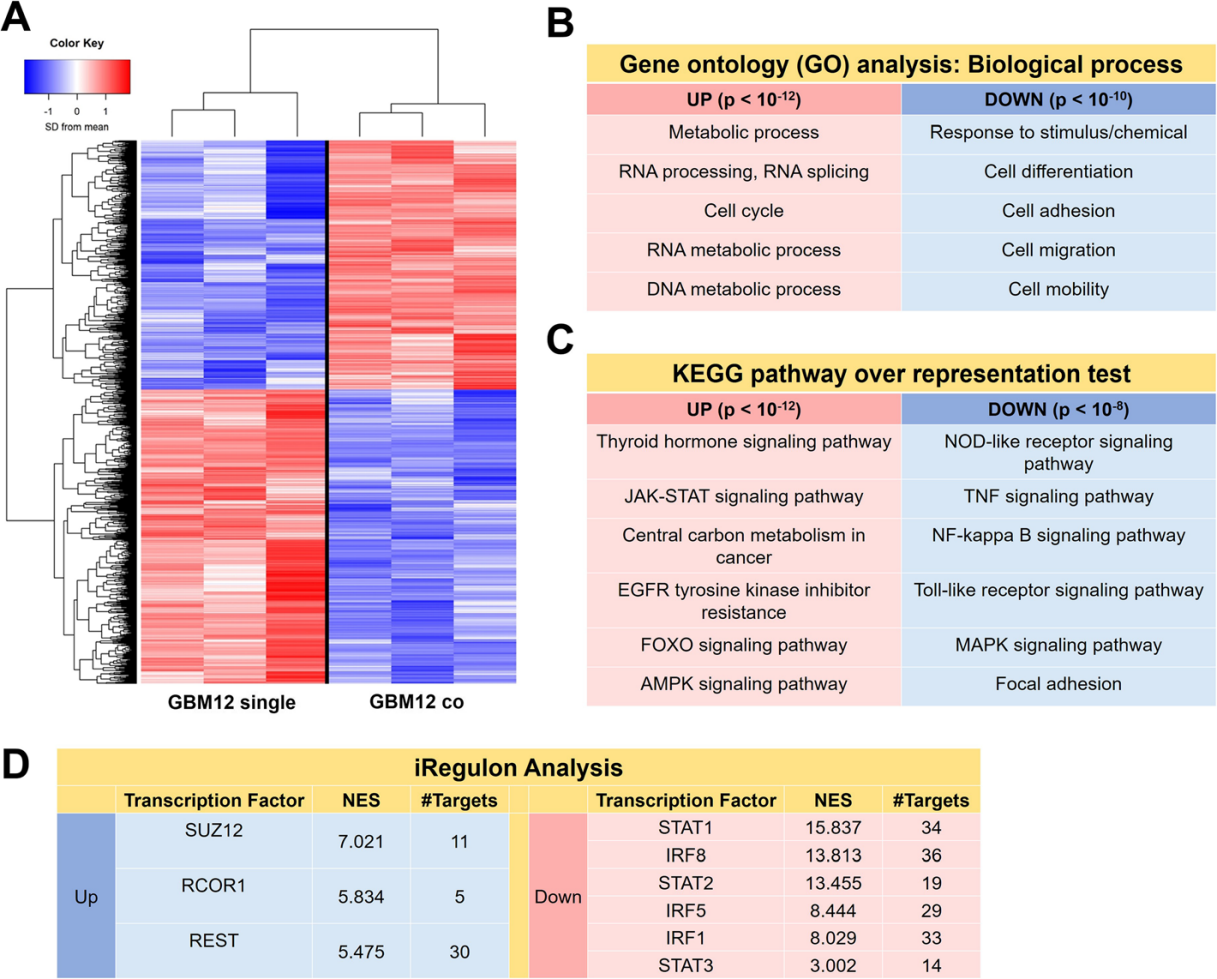
Soluble signals from microglia induced shifts in GBM12 cell transcriptome. **a** RNAseq and differential gene expression analysis (*p* < 0.05) identified 1563 upregulated genes and 1846 downregulated genes in GBM12 co compared to GBM12 single. Selected (**b**) gene ontology (GO) and (**c**) KEGG pathway terms suggest GBM-MG interactions underlie upregulated expression of genes involved in GBM proliferation and genes with diminished expression associated with GBM invasion. (*n* = 3 for GBM12 single and GBM12 co) (**d**) Significant putative genes from the top 50 genes with normalized enrichment score > 3 were identified via iRegulon

RNAseq analysis was used to examine global shifts in the transcriptomic profile of GBM12 specimens as a result of MG crosstalk. Two RNAseq datasets were compared: (1) a *co-culture specimen* containing RNA isolated from the GBM-seeded hydrogels kept in physically separated co-culture with HMC3 MG-seeded hydrogels; and (2) a *GBM single* culture specimen containing RNA isolated from GBM-seeded hydrogels cultured independently (*n* = 3 for each group). This comparison allowed investigation of global shifts in GBM gene expression profiles only due to GBM-MG crosstalk (Fig. 3). Differential gene expression (DE) analysis was performed using an FDR-adjusted *p* value set to 0.05. In total, there were 3409 DE genes, of which 1563 were upregulated and 1846 were downregulated as a result of co-culturing GBM cells with microglia. Gene ontology (GO) analysis for biological process ontology showed upregulated genes were associated with proliferation and RNA/DNA replication and proliferation, while downregulated genes were associated with motility, adhesion, and invasion (Supplementary Table S1; Fig. 3b).

KEGG pathway analysis was used to identify the top 20 up- and downregulated pathways (Table S2; Fig. 3c). GBM-MG *co-culture* downregulated multiple immune response-related pathways in GBM cells including nucleotide-binding and oligomerization domain (*NOD*)-like receptors (*NLRs*), tumor necrosis factor (*TNF*) signaling, nuclear factor kappa-beta (*NF-κB*) signaling, toll-like receptor signaling (*TLRs*), and mitogen-activated protein kinase (*MAPK*) signaling. GBM-MG co-culture decreased focal adhesion pathway activation. Upregulated KEGG pathways included those involved in cell proliferation and survival included thyroid hormone signaling, Janus kinase-signal transducer and activator of transcription pathway (*JAK-STAT*) signaling, central carbon metabolism, forkhead box transcription factors (*FOXO*) signaling, and adenosine monophosphate-activated protein kinase (*AMPK*) signaling. Intriguingly, *EGFR* tyrosine kinase inhibitor resistance was also increased, indicating that the co-culture might also alter the therapeutic response to classes of drugs targeting receptor tyrosine kinase inhibitors. iRegulon analysis of upregulated genes showed high normalized enrichment scores for *SUZ12* (NES = 7.021), *RCOR1* (NES = 5.834), and *REST* (NES = 5.475). iRegulon analysis also showed high normalized enrichment scores for downregulated genes *STAT1/2/3* (NES = 15.831/13.455/3.002) and *IRF1/5/8* (NES = 8.029, 8.440, 13.813). Together, these analyses indicate that co-culture with microglia induces significant shifts in GBM transcriptome linked to increased proliferative behavior and decreased invasive potential.

### Microglial soluble factors inhibit GBM invasion

**Fig. 4:**
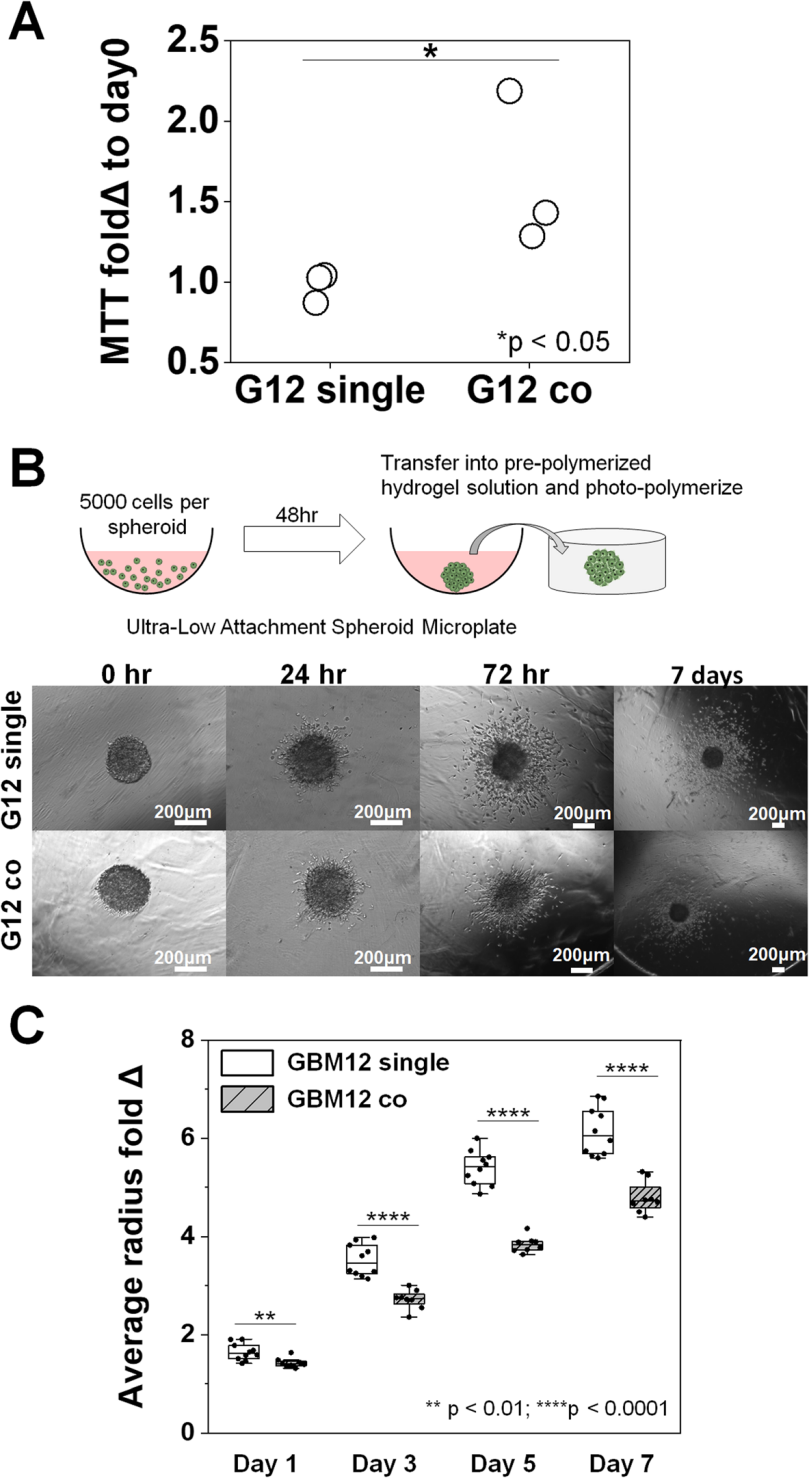
Soluble factors from microglia increase GBM proliferation but significantly inhibit GBM12 invasion. **a** Significant increase in MTT fold change at day 3 in GBM12 cells co-cultured with MG compared to GBM12 single cultures (both *n* = 3). **b** Representative images of spheroid-based invasion assay for GBM12 single cultures and GBM12-microglia co-cultures. Scale: 200 μm. **c** Quantification of GBM cell invasion in the presence or absence of soluble signals from microglia, with results reported as the mean radius of the invasion front of GBM cells versus the starting radius of the spheroid. GBM12 cells exhibited significantly decreased invasion (*n* = 8) in response to co-culture with HMC3 microglia (vs. GBM12 alone; *n* = 10). *p < 0.05, **p < 0.01, ****p < 0.0001

**Fig. 5:**
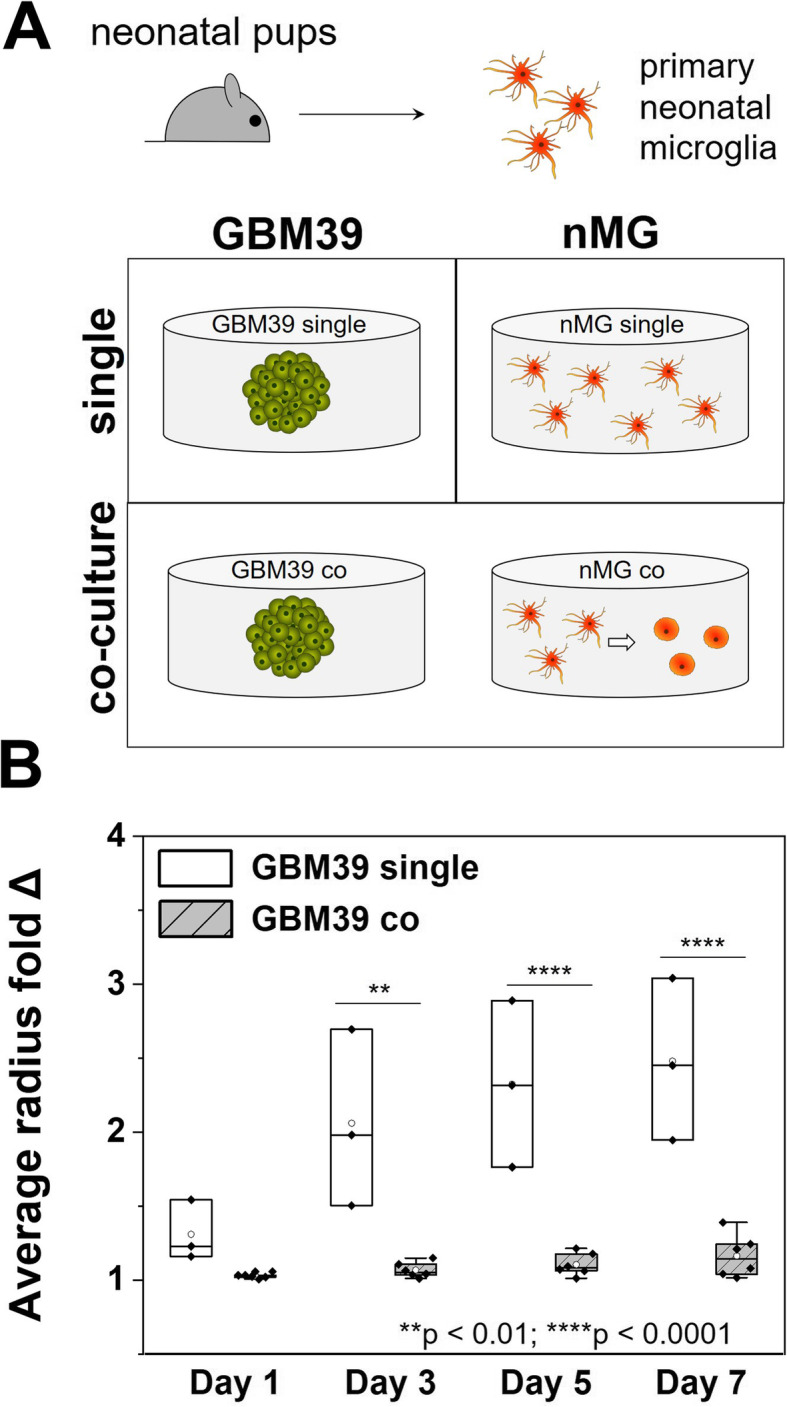
Primary neonatal microglia strongly inhibit GBM invasion. **a** Primary neonatal microglia cells (nMG) were obtained from neonatal mouse pups. nMG and GBM39 seeded hydrogel disks were then either cultured alone (single) or together (co) in the same well of a 24-well plate. **b** GBM39 invasion was significantly decreased due to co-culture with nMG (*n* = 6) compared to GBM39 alone (*n* = 3). **p < 0.01, ****p < 0.0001

GBM proliferation and invasion was subsequently examined as a function of MG co-culture. The proliferative activity of GBM12 cells, reported as a fold change increase in MTT activity (MTT of GBM cells at day 3 vs. MTT of the same cultures at day 0), increased significantly in response to GBM-MG co-culture (Fig. 4a). MG *co-culture* strongly inhibited GBM12 invasion over the course of a seven-day spheroid-based invasion assay, with effects observed as early as after 24 h (Fig. 4b, c). A consistent inhibitory effect of MG co-culture on GBM invasion was observed as well for a different source of microglia (primary neonatal microglia, nMG) and a different patient-derived GBM cell population (GBM39: *EGFR*^*VIII*^). GBM39 show reduced invasive potential in vivo compared to GBM12, but nonetheless GBM39 invasion was again significantly inhibited in the multi-dimensional hydrogel model in response to nMG co-culture, with effects observed as early as day 3 (Fig. 5).

### Profiling MG-GBM secretome using cytokine array

**Fig. 6:**
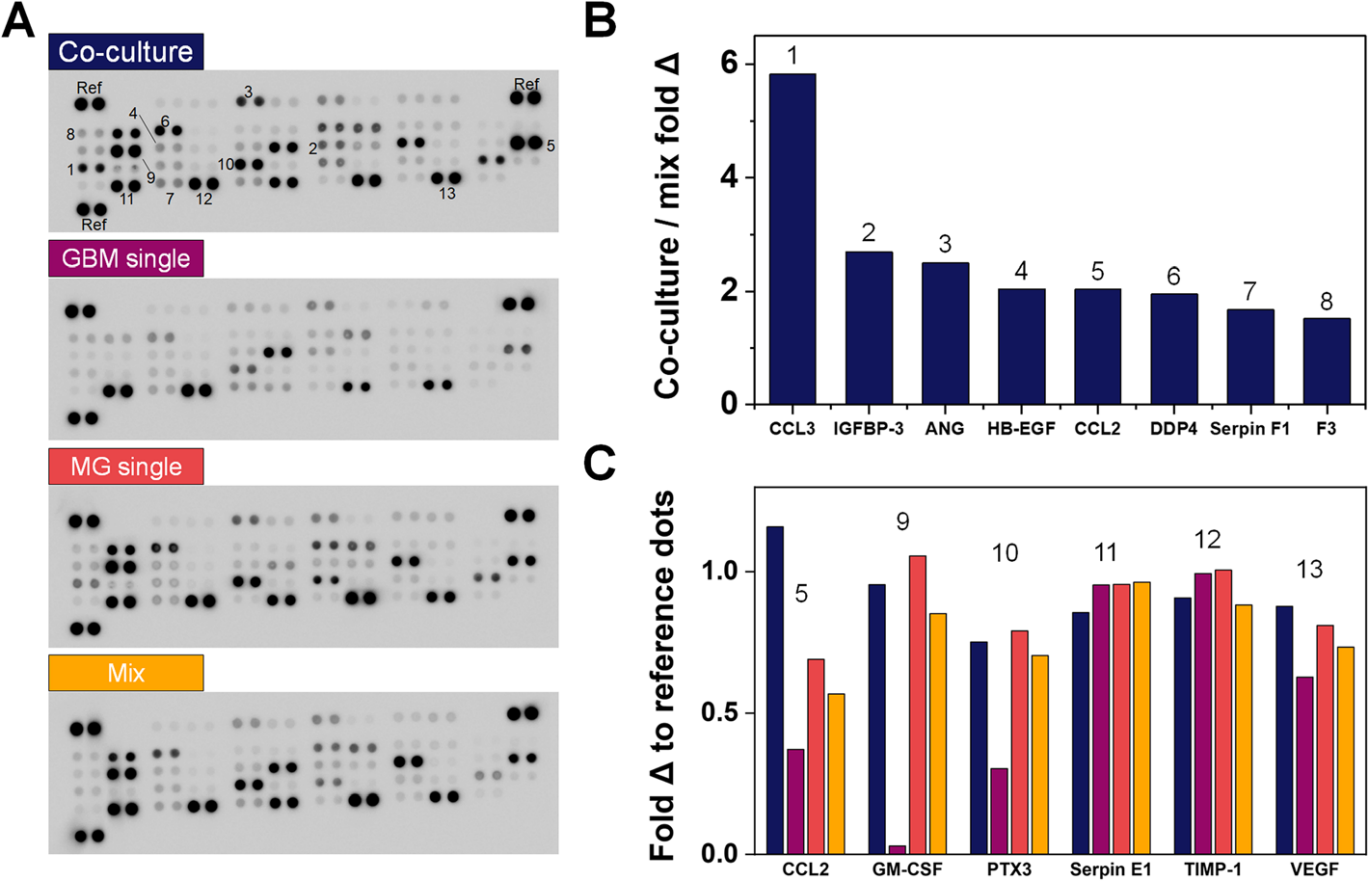
Profiling GBM-MG (GBM12-HMC3) secretome linked to decreased GBM cell invasion. **a** Proteome Profiler^TM^ Array analysis of secretome profiles from conditioned media of 4 distinct culture conditions: GBM-MG *co-culture*; *GBM single*; *MG single*; 1:1 *Mix* of *GBM single*; and *MG single*. **b** Eight factors that displayed > 1.5 fold change in GBM-MG *co-culture* versus *Mix* media groups. **c** Six factors (from GBM-MG *co-culture*) that displayed greater than 0.75-fold change versus the positive reference dots. Numbers (1–13) correspond to positions labeled on blots in **a**. Raw data are available in Table S3

A secretome screen was performed to compare cell culture media from GBM-MG *co-culture* versus GBM or MG monocultures (*GBM single*, *MG single*) and a *Mix* media (1:1 mixture of *GBM single* and *MG single* medias; Fig. 6; raw data are available in Table S3). Eight factors exhibited a > 1.5 fold change in *co-culture* vs. single cultures: chemokine ligand 2 and 3 (CCL2, CCL3); insulin-like growth factor binding protein 3 (IGFBP-3); angiogenin (ANG); heparin-binding epidermal growth factor-like growth factor (HB-EGF); dipeptidyl peptidase-4 (DPP4); Serpin F1; and coagulation factor III (F3). Six factors were expressed at levels greater than 0.75 of the positive reference intensity value within each secretome array (Fig. 6c). Of these, CCL2 showed the largest activation (> 2-fold change) for GBM-MG *co-culture* versus the *Mix* media control. Granulocyte-macrophage colony-stimulating factor (GM-CSF, or CSF2) and pentraxin-related protein (PTX3) were both highly expressed in cultures containing MG (*MG-single*, *Mix*, *co-culture*) media but lowly expressed in *GBM-single* hydrogels. Serpin E1, tissue inhibitor of metalloproteinase-1 (TIMP-1), and vascular endothelial growth factor (VEGF) were expressed across all culture conditions.

## Discussion

Cellular crosstalk within the tumor microenvironment provides a powerful avenue of interaction that may significantly shape disease progression. Tools to interrogate cellular crosstalk offer an opportunity to identify novel therapeutic compounds to improve treatment of glioblastoma. This work demonstrates the use of a tissue engineering platform to investigate the role of crosstalk between patient-derived GBM specimens and microglia on shifts in the phenotypic, proteomic, and transcriptomic signatures of GBM. GBM-MG crosstalk is bidirectional and induces microglia activation along with shifts in GBM cell activity consistent with the go-or-grow phenomenon [61]. This work extends technical capabilities beyond more traditional Transwell membrane or mixed culture methods for examining cell-cell crosstalk. Importantly, this effort provides a platform for analysis of individual cell populations, each maintained within discrete multi-dimensional models of the tumor microenvironment, while maintaining the ability to examine the nature and role of cell-cell crosstalk during mixed culture on cell activity.

While microglia display a quiescent phenotype in single culture, hallmarks of microglial activation were observed via *both* morphological changes and increased CD68 expression as a result of GBM-MG co-culture. These results are consistent with hallmarks of microglial activation seen in cases of disease and histopathological analysis of GBM tumors [24, 62]. The nature of this tissue engineering platform allows significant post-culture analysis of GBM cell activity at functional (invasion, proliferation), transcriptomic (RNAseq), and secretomic levels. We recently reported the use of gelatin hydrogels to profile to role of localized hypoxia on activation of cells associated with the neurovascular unit [58]. Indeed, while a single hydrogel formulation was used for all microglial culture in this study, significant opportunities exist to use multi-dimensional hydrogels culture to refine our understanding of the role of the matrix microenvironment on microglia activation itself.

While advanced sequencing techniques such as RNA sequencing offer the opportunity to define the transcriptomic signature of cells to aid treatment planning and outcome prediction [40, 43, 44, 63], the design of the hydrogel culture system reported here enabled analysis of shifts in the transcriptome of individual cell populations as a result of heterotypic cell (GBM, MG) crosstalk. GO analyses revealed GBM-MG *co-culture* upregulated genes in a patient-derived GBM specimen associated with cell cycle, RNA/DNA division and metabolic activity. However, genes involved in cell adhesion/migration showed significant downregulation as a result of GBM-MG *co-culture*. These findings indicate tradeoffs in GBM proliferation versus invasion due to MG crosstalk consistent with the go-or-grow dichotomy of GBM cells [32, 61, 64, 65]. Significant decreases were observed in expression of genes associated with *NLR*, *TNF*, *NF-κB*, *MAPK*, and *TLR* pathways in GBM specimens in response to MG *co-culture*. *NLR* and *TLR* signaling pathways are involved in pathophysiological responses to inflammation and tumor progression [66–68]. Of these, the *NF-κB* signaling pathway is known to be sensitive to *TNF* signaling [69–71] which plays a major role in immune activation [72, 73], breast cancer invasion [74], and driving *TLR* and *MAPK* signaling involved in cell migration and tumor invasion. These pathways contribute to heightened immune responsiveness and are involved in angiogenesis and cell migration [66, 67, 69–71], suggesting GBM-MG interactions may inhibit GBM invasiveness.

KEGG analysis also showed strong upregulation in *TH* and *STAT3* signaling, indicating that secreted factors from microglia may promote GBM proliferation, reduce apoptosis, and enhance chemotherapeutic resistance [75–77]. Recently, our group showed STAT3 is strongly activated in GBM, and inhibiting STAT3 can reduce GBM cell proliferation [52, 78]. More, GBM-MG *co-culture* upregulated *FOXO* signaling, which has been linked to therapeutic resistance due to its contribution to DNA repair as well as mediation of oxidative stress. iRegulon analysis showed GBM-MG crosstalk increased enrichment for *SUZ12,* previously shown to be increased in high grade astrocytoma and involved in pathways that regulate glioma proliferation and metastasis [79, 80]. iRegulon analysis also showed GBM-MG crosstalk increased *REST* and *RCOR1*, known to regulate the oncogenic properties of GBM stem cells [81] that associate with therapeutic resistance and recurrence [82, 83]. The *IRF* family has been shown to be significant tumor suppressors, inhibit tumor proliferation and loss of *IRF* genes may contribute to tumor metastasis and invasion [84, 85]. Together, analysis of transcriptomic data support the functional responses of increased proliferation but decreased invasion for GBM cells as a result of GBM-MG interactions [32, 65, 86–88]. Inclusion of RNAseq analyses to examine the role of microglia-GBM crosstalk provides a valuable dataset to motivate ongoing efforts. Indeed, while this study highlighted the importance of examining GBM-microglia interactions via RNAseq methods, ongoing efforts are using this approach to consider the role of microglia signaling on GBM subtractions such as glioblastoma stem cells (GSCs) as well as examining the behavior of GSCs within a larger cohort of GBM cells.

The hydrogel platform was subsequently used to experimentally interrogate the influence of GBM-MG crosstalk on GBM proliferative and invasive phenotypes in patient-derived GBM12 cells. GBM12 cells exhibited significantly increased proliferation and significantly inhibited invasion in response to MG co-culture. Strikingly, MG-induced inhibition of GBM12 invasion was observed for multiple combinations of patient-derived GBM specimens and microglia: *EGFR*^*OE*^ GBM12 cells co-cultured with HMC3 microglia and *EGFR*^*vIII*^ GBM39 cells co-cultured with primary mouse neonatal microglia.

Analysis of the combined GBM-MG secretome revealed multiple targets driving the observed shifts in functional and transcriptomic activity. CCL2 and CCL3 are associated with monocyte and macrophage recruitment [89–91] and may act as chemoattractant [89]. Of these, further study of the role of CCL2 in GBM invasion may be particularly warranted, as expression levels were not only significantly increased in GBM-MG *co-culture* (vs. *GBM-single* or *MG-single* cultures) but also compared to the *Mix* control, consistent with synergistic activation of CCL2 secretion due to GBM-MG crosstalk. IGFBP-3, known to regulate cell proliferation, was also increased in GBM-MG crosstalk, though its role in cancer progression remains to be fully understood [92–94]. DPP4 (plasma membrane protein that contributes to immune and metabolic regulation [95, 96]) and HB-EGF (cell metabolic activity and tumor suppression in other cancers [95–97]) were also strongly upregulated in GBM-MG *co-culture*, as was ANG, well-known for its role in angiogenesis and cell proliferation [98, 99], and Serpin F1, known as for its role in suppression of tumor growth and prostate cancer metastasis [100–103]. Previous study by Shinozaki et al. [104] also indicated that cytokines produced by microglia could potentially drive astrocytes towards a neuroprotective phenotype upon brain injuries. While results here provide critical data regarding highly expressed factors within the combined MG-GBM secretome, ongoing opportunities exist to consider a wider range of secreted factors, consider the role of alternative signaling pathways such as extracellular vesicles in crosstalk, and to use machine learning algorithms to identify critical subsets of factors most highly associated with GBM cell response. Notably, we recently reported an iterative partial least squares regression machine learning methods to identify [105] secretome signals generated by niche-associated cells that enhance quiescence of hematopoietic stem cells in hydrogel culture. So, while further efforts are needed to more fully investigate the potential mediators of GBM invasion that arise from GBM-MG crosstalk, we present a robust platform to pursue such investigations here.

The immune system and immune cells and their relationship with cancer have been a hot topic in recent years. Tumor-associated macrophages/microglia, or GBM-associated macrophages/microglia here, has drawn a large amount or research efforts [19–21]. While some studies showed that the infiltrated microglia facilitates the tumor growth and targeting those infiltrated immunes cells could be a promising therapeutic approach [19–21, 106], the exact role of them remains controversial [107]. In the study, the combination of increased proliferation but decreased invasion aligns with the go-or-grow hypothesis [64], but more importantly demonstrates that crosstalk between MG and GBM cells in the tumor microenvironment may have powerful effects on GBM activities tied directly to tumor progression and patient survival.

Finally, we note the value and limits of a tissue engineering approach described herein. Glioblastoma tumors contain a heterogeneous mix of cells, including a subpopulation of tumor initiating cells (GBM stem cells, GSCs) [108–110], critical for invasion, recurrence, and mortality [109, 111–117]. Tumors contain a mix of fibrillar matrix (e.g*.*, collagens, laminins) and hyaluronic acid (HA), complex perivascular niches, regions of hypoxia [118], and multiple immune-associated cells including microglia and macrophages. Here, we report a multi-dimensional hydrogel platform to examine pathophysiological processes linked to GBM progression and mortality using patient-derived GBM specimens in response to microglia. We have also recently described hydrogel-based platforms to investigate the role of angiocrine signaling from engineered perivascular cultures on GBM cell invasion and resistance to the frontline chemotherapy temozolomide [57]. We also reported adaptations to the hydrogel environment via localized formation of hypoxic zone to support culture of a broader diversity of cells from the neurovascular unit [58]. While we acknowledge understanding the role of the coordinated activity of tumor-associated macrophages and microglia on the activity of GBM cell cohorts (or on specific cells from the GBM microenvironment such as GSCs) are essential, this manuscript provides a conceptual framework for pursuing such studies while also providing critical information regarding the role of reciprocal GBM-microglia signaling on GBM invasion.

## Conclusion

This study describes a tissue engineering platform to examine the role of GBM-microglia crosstalk on processes associated with GBM progression. It also shows the ability to use bioinformatic tools to identify transcriptomic shifts underlying these responses. We show dynamic, two-way interactions between patient-derived GBM cells and microglia via paracrine signaling influence both microglia and GBM cell phenotype. Microglia in the presence of patient-derived GBM cells showed morphological shifts associated with activation. Microglia co-culture significantly inhibited GBM invasion but enhanced proliferation that could be captured via three-dimensional spheroid invasion assays and transcriptomic analyses. Future efforts will seek to understand the contribution of GBM-microglia crosstalk on tumor resistance to therapeutics, to reveal candidate signaling axes for rational combinatorial targeting.

## Supplementary information

Additional file 1: **Figure S1.** Raw images for β-actin Western blot samples (left three: MG single; right three: MG co). Figure S2. Raw images for CD68 Western blot samples (left three: MG single; right three: MG co). Table S1. Selected gene ontology (GO) biological process terms that showed significant differences in overrepresented test of GBM co-culture compared to single culture. Table S2. Kyoto Encyclopedia of Genes and Genomes (KEGG) pathways that showed significant differences in overrepresented test of GBM co-culture compared to single culture. Table S3. Secretome profiling pixel intensities for individual blots.

## Data Availability

Data and metadata associated with this manuscript are available on request to the corresponding author.

## Abbreviations

AMPK: Adenosine monophosphate-activated protein kinase
ANG: Angiogenin
CCL: Chemokine ligand
CNS: Central nervous system
DE: Differential gene expression
DPP4: Dipeptidyl peptidase-4
EGFR: Epidermal growth factor receptor
F3: Coagulation factor III
FDR: False discovery rate
FOXO: Forkhead box transcription factors
GBM: Glioblastoma
GelMA: Gelatin methacrylate
GM-CSF: Granulocyte-macrophage colony-stimulating factor
GO: Gene ontology
HB-EGF: Heparin-binding epidermal growth factor-like growth factor
IGFBP: Insulin-like growth factor binding protein
IRF: Interferon regulatory factor
JAK: Janus kinase
KEGG: Kyoto Encyclopedia of Genes and Genomes
MAPK: Mitogen-activated protein kinase
MG: Microglia
NES: Normalized enrichment score
NLRs: Nucleotide-binding and oligomerization domain-like receptors
NF-κB: Nuclear factor kappa-beta
PDC: Patient-derived GBM cell
PTX3: Pentraxin-related protein
RIN: RNA integrity number
RNAseq: RNA sequencing
STAT: Signal transducer and activator of transcription
TIMP: Tissue inhibitor of metalloproteinase
TLRs: Toll-like receptor signaling
TNF: Tumor necrosis factor
VEGF: Vascular endothelial growth factor
